# Bioenergetic state regulates innate inflammatory responses through the transcriptional co-repressor CtBP

**DOI:** 10.1038/s41467-017-00707-0

**Published:** 2017-09-22

**Authors:** Yiguo Shen, David Kapfhamer, Angela M. Minnella, Ji-Eun Kim, Seok Joon Won, Yanting Chen, Yong Huang, Ley Hian Low, Stephen M. Massa, Raymond A. Swanson

**Affiliations:** 1 Department of Neurology, University of California, San Francisco, CA 94143 USA; 2 Neurology Service, San Francisco Veteran Affairs Medical Center, 4150 Clement St., San Francisco, CA 94121 USA; 3 Department of Neurology, Affiliated Drum Tower Hospital, Nanjing Medical University, Jiangsu, 210008 People’s Republic of China; 4 Present Address: Adaptive Biotechnologies, 329 Oyster Point Blvd., South San Francisco, CA 94080 USA

## Abstract

The innate inflammatory response contributes to secondary injury in brain trauma and other disorders. Metabolic factors such as caloric restriction, ketogenic diet, and hyperglycemia influence the inflammatory response, but how this occurs is unclear. Here, we show that glucose metabolism regulates pro-inflammatory NF-κB transcriptional activity through effects on the cytosolic NADH:NAD^+^ ratio and the NAD(H) sensitive transcriptional co-repressor CtBP. Reduced glucose availability reduces the NADH:NAD^+^ ratio, NF-κB transcriptional activity, and pro-inflammatory gene expression in macrophages and microglia. These effects are inhibited by forced elevation of NADH, reduced expression of CtBP, or transfection with an NAD(H) insensitive CtBP, and are replicated by a synthetic peptide that inhibits CtBP dimerization. Changes in the NADH:NAD^+^ ratio regulate CtBP binding to the acetyltransferase p300, and regulate binding of p300 and the transcription factor NF-κB to pro-inflammatory gene promoters. These findings identify a mechanism by which alterations in cellular glucose metabolism can influence cellular inflammatory responses.

## Abstract (web summary)

Several metabolic factors affect cellular glucose metabolism as well as the innate inflammatory response. Here, the authors show that glucose metabolism regulates pro-inflammatory responses through effects on the cytosolic NADH:NAD+ ratio and the NAD(H)-sensitive transcription co-repressor CtBP.

## Introduction

Microglia and macrophages are innate immune responders to infection, tissue damage, and other stressors. The early phase of this response involves release of pro-inflammatory cytokines, nitric oxide, and metalloproteinases, along with alterations in cell morphology and surface protein expression^1, 2^. Although these responses are adaptive in the setting of infection, they can be deleterious in non-infectious injuries such as stroke and head trauma. Accordingly, factors that suppress the acute inflammatory reaction also reduce tissue loss and improve functional outcomes in animal models of non-infectious brain injury^3, 4^.

Inflammatory responses are influenced at the transcriptional level by factors that affect cellular bioenergetic state, such as caloric restriction, ketogenic diet, and the glycolytic inhibitor 2-deoxyglucose (2DG)^5, 6^. Caloric restriction, ketogenic diet, and 2DG each produce a ketogenic state in which glucose utilization is suppressed, and these conditions also reduce brain inflammation, tissue loss, and functional impairment after brain injury^7–12^. Conversely, hypoxia and hyperglycemia promote glucose utilization, exacerbate inflammation, and worsen outcomes after brain injury^13, 14^.

How inflammation affects cell metabolism is well established^15^, but less is known about how energy metabolism affects inflammatory responses. One potential mechanism is through the cytosolic NADH:NAD^+^ ratio, which is thermodynamically coupled to glycolysis. The cytosolic NADH:NAD^+^ ratio is decreased by ketogenic factors such as dietary restriction and 2DG, which decrease flux through glycolysis, and increased by conditions such as hypoxia and hyperglycemia, which increase flux through glycolysis. Cytosolic NADH and NAD^+^ are physically separated from the mitochondrial metabolite pools, but can move freely across the nuclear membrane to influence transcriptional events.

Despite the fundamental roles of NAD^+^ and NADH in cell metabolism, only a limited number of NADH-sensitive proteins are known to affect gene transcription^16^. Among these are the C-terminal binding proteins (CtBP), which function as transcriptional corepressors. Mammals express two CtBP proteins, CtBP1 and CtBP2, that exhibit overlapping actions^17, 18^. CtBP forms repressor complexes with histone deacetylases, histone methyl transferases, E3 ligases, and other transcriptional regulators^19, 20^. Some of these complexes include CtBP homodimers or heterodimers, while others require CtBP in its monomeric form^21–24^. CtBP in its monomeric form suppresses the activity of the acetyltransferase p300/CBP^20^, which acetylates both histones and the pro-inflammatory transcription factor, NF-κB^25^. Increased NADH levels promote the formation of CtBP dimers and higher order oligomers, and thereby modulate CtBP association with its binding partners^26^.

Here, we investigate whether anti-inflammatory effects of ketogenic metabolism may be mediated through an NADH/CtBP signaling mechanism. We show that changes in cytosolic NADH:NAD^+^ ratio influence inflammatory responses by regulating NF-κB transcriptional activity through a mechanism requiring CtBP dimerization. This process involves dissociation of p300 from CtBP and acetylation of the NF-κB p65 subunit.

## Results

### Microglial and macrophage activation is suppressed by 2DG

**Fig. 1:**
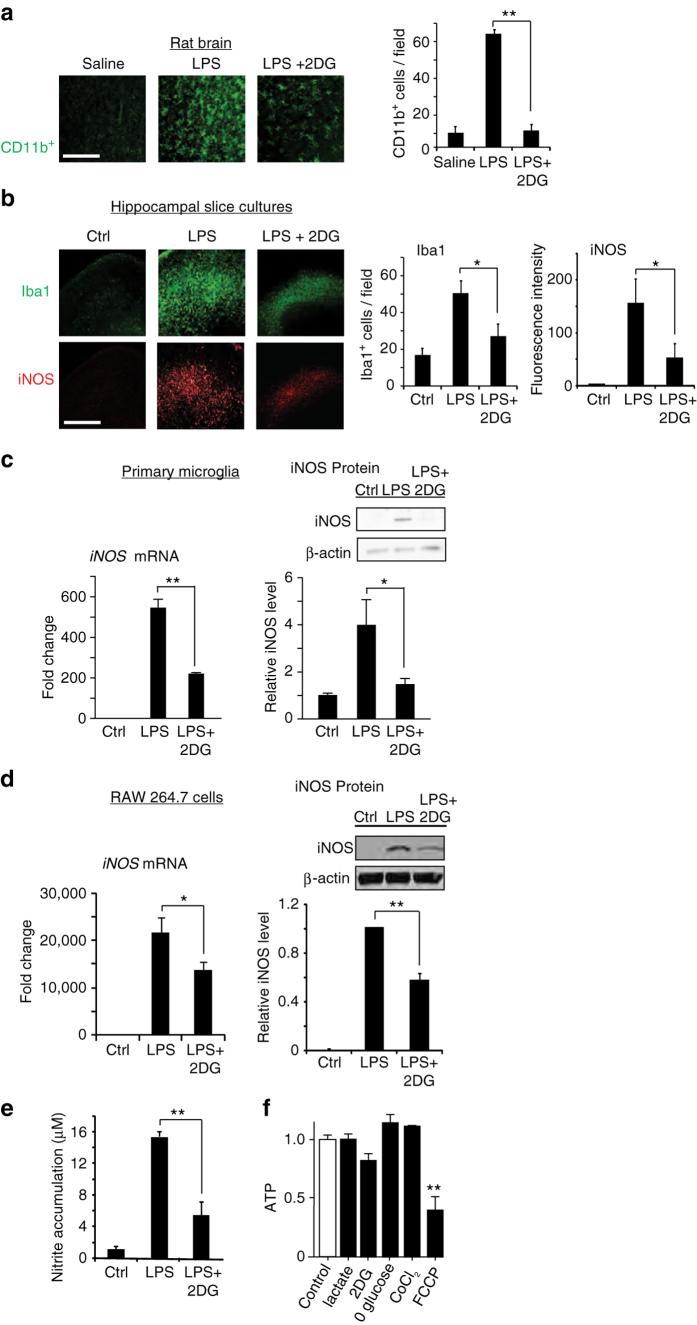
2-deoxyglucose suppresses LPS-induced microglial activation. **a** Immunostaining for CD11b identifies activated microglia in rat hippocampus. CD11b expression was increased 24 h after intraperitoneal injection with LPS (10 mg/kg). The increase was attenuated by co-injection with 2-deoxyglucose (2DG; 100 mg/kg). *Scale bar* = 100 µm. **p < 0.01, *n* = 6. **b** Immunostaining for Iba1 and iNOS identify activated microglia in mouse hippocampal slice cultures after 24 h incubation with LPS (10 μg/ml) or LPS + 2DG (1 mM). *Scale bar* = 100 µm; *n* ≥ 3, *p < 0.05. Culture medium contained 6 mM glutamine and 5 mM glucose. **c** Effects of 1 mM 2DG on LPS (10 ng/ml)—induced iNOS transcript and protein expression in primary microglial cultures. *n* = 5; *p < 0.05, **p < 0.01. Full length immunoblots are shown in Supplementary Fig. 3. **d**, **e**. Effects of 1 mM 2DG on LPS-induced iNOS protein expression, mRNA expression, and nitric oxide production in cultured RAW267.4 cells. *n* ≥ 3; *p < 0.05, **p < 0.01. **f** Relative ATP levels measured after 24 h incubation in control medium, 1 mM 2-deoxyglucose, 200 µm CoCl_2_, 20 mM lactate, glucose-free medium, or in 2 µm trifluorocarbonylcyanide phenylhydrazone (FCCP) as a positive control. *n* = 4; **p < 0.01 v. control. *Error bars* show s.e.m

The reduced glycolytic flux resulting from caloric restriction and ketogenic diet can be mimicked by the glycolytic inhibitor 2DG^27, 28^. To determine whether 2DG can replicate the effect of ketogenic diet on brain inflammatory responses, we treated rats with intraperitoneal injections of lipopolysaccharide (LPS) or with LPS + 2DG. The systemic LPS injection induced a robust activation of brain microglia, and this was strikingly reduced by co-administration of 2DG (Fig. 1a). In organotypic brain slice cultures (Fig. 1b), 2DG likewise suppressed the effect of LPS on microglial activation and on the expression of inducible nitric oxide synthase (iNOS), a hallmark of inflammatory activation in microglia and macrophages^29^. Primary microglial cultures similarly showed an attenuated response to LPS in the presence of 2DG (Fig. 1c), thus confirming that the effects of LPS and 2DG on microglia are not dependent upon indirect, systemic effects of these agents. This same pattern of responses was observed in the RAW264.7 macrophage cell line. RAW264.7 cells treated with LPS showed increased iNOS mRNA expression, iNOS protein expression, and nitric oxide production, all of which were attenuated by co-incubation with 2DG (Fig. 1d, e). RAW264.7 cells were used because, unlike primary microglia, they can be efficiently transfected and are not readily activated by transfection procedures. Glutamine was provided as an alternative energy substrate in all of the in vitro studies, as glutamine is taken up by cells and metabolized to the keto acid, α-ketoglutarate. ATP measurements confirmed no significant effect of 2DG or glucose-free medium on ATP levels in the glutamine-supplemented cell cultures (Fig. 1f).

### Glycolytic inhibition reduces cytosolic NADH:NAD^+^ ratio

**Fig. 2:**
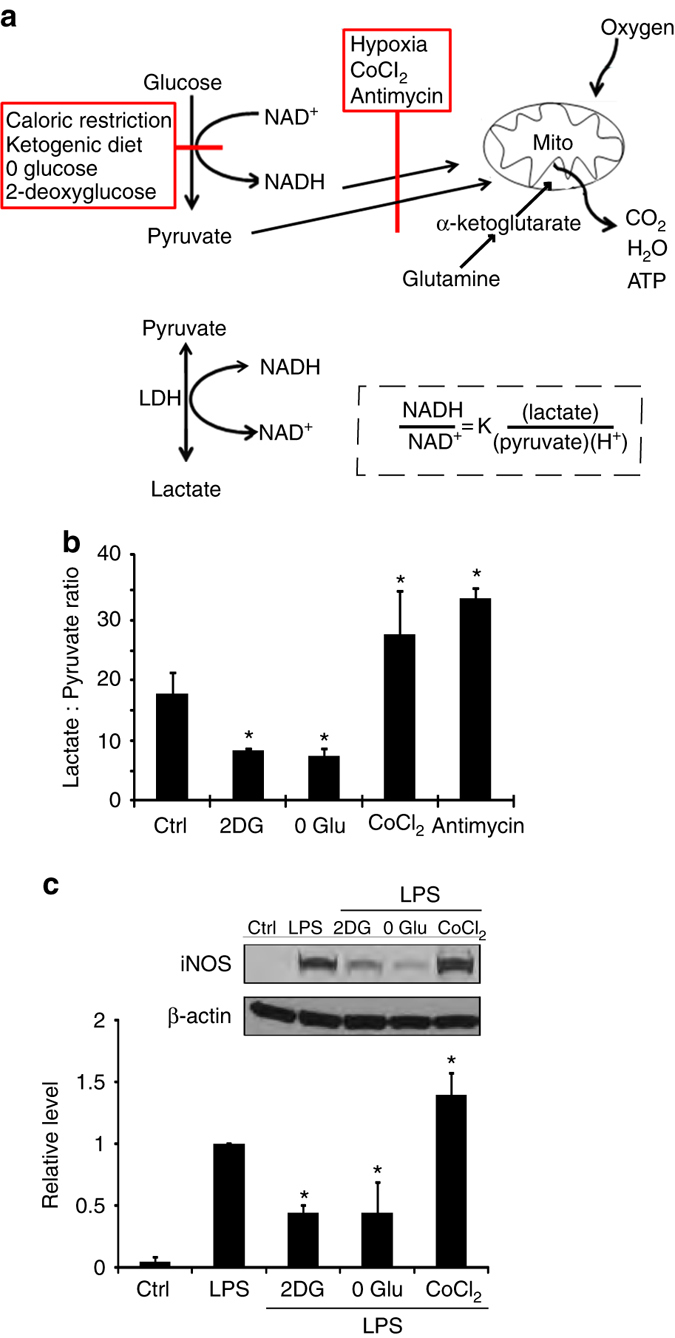
Relationships between glucose metabolism and cytosolic NADH:NAD^+^ ratio. **a** Factors that reduce glucose flux through glycolysis, such as reduced glucose availability or glycolytic inhibitors, reduce NADH levels and thereby reduce NADH:NAD^+^ ratio, whereas factors that inhibit oxidative metabolism, such as hypoxia and mitochondrial inhibitors, have the opposite effect. Glutamine provides ketone bodies (α-ketoglutarate) to fuel mitochondrial ATP production in the absence of glycolysis. Lactate dehydrogenase (LDH) maintains the lactate:pyruvate ratio in equilibrium with the cytosolic NADH:NAD^+^ ratio. **b** The lactate:pyruvate ratio provides an index of the cytosolic NADH:NAD^+^ ratio in cells treated with glycolytic and mitochondrial inhibitors. 2DG, 1 mM 2-deoxyglucose; 0 Glu, glucose-free medium; CoCl_2_, 200 µm cobalt chloride; antimycin, 1 µm antimycin A. *n* = 4; *p < 0.05 v. control. **c** LPS-induced iNOS expression was suppressed in RAW267.4 cells treated with 1 mM 2DG or glucose-free medium, and increased in cells treated with the mitochondrial inhibitor cobalt chloride (CoCl_2,_ 200 µm). *n* = 4; *p < 0.05 v. control. *Error bars* show s.e.m. Full length immunoblots are shown in Supplementary Fig. 3

We assessed the free cytosolic NADH:NAD^+^ ratio by measuring the cellular lactate:pyruvate ratio^30^. This indirect approach has advantages over direct biochemical measurements in that it is not influenced by protein bound or mitochondrial NAD^+^ and NADH^30, 31^. 2DG and glucose-free medium are predicted to lower the cytosolic (i.e., non-mitochondrial) NADH:NAD^+^ ratio by decreasing glucose flux through glycolysis, while impaired respiration should increase glycolytic flux and elevate the cytosolic NADH:NAD^+^ ratio (Fig. 2a). As expected, both 2DG and glucose-free medium reduced the lactate:pyruvate ratio (indicating a reduced free cytosolic NADH:NAD^+^ ratio), while the respiratory inhibitors antimycin A and cobalt chloride (CoCl_2_)^27, 32^ increased it (Fig. 2b). We then evaluated these treatments on LPS-induced iNOS expression. Glucose-free medium suppressed LPS-induced iNOS expression to an extent comparable to that seen with 2DG, while CoCl_2_ had the opposite effect (Fig. 2c). These findings identify a correlation between changes in the inflammatory response and changes in the NADH:NAD^+^ ratio in response to metabolic factors.

### Elevated NADH reverses the anti-inflammatory effect of 2DG

**Fig. 3:**
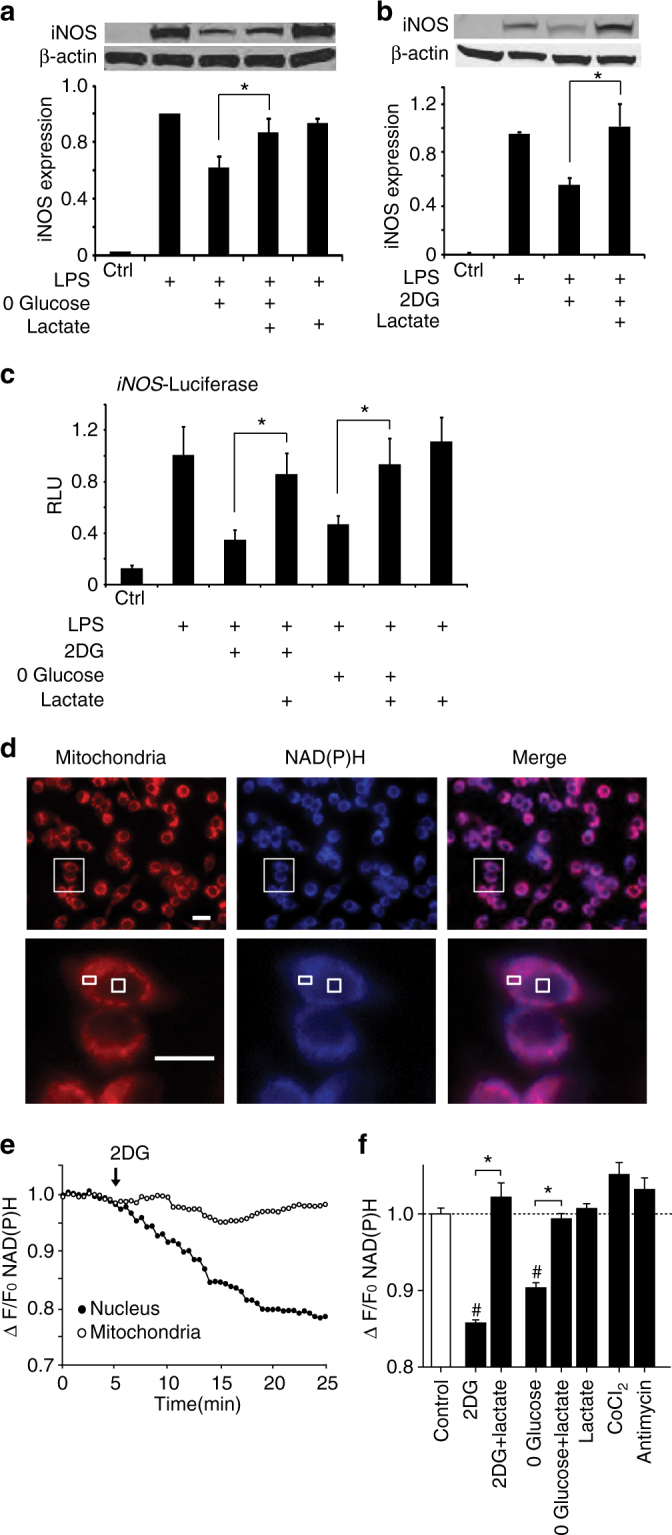
Lactate reverses the effects of glycolytic inhibition on both cytosolic NADH levels and LPS-induced iNOS expression. **a**, **b** The effects of glucose-free medium and 2DG on LPS-induced iNOS expression are reversed by 20 mM lactate. *n* > 3, *p < 0.05. Full length immunoblots are shown in Supplementary Fig. 3. **c** The effects of glucose-free medium and 2DG on LPS-induced *iNOS* transcription are reversed by 20 mM lactate, as measured by relative light units (RLU) emitted by a cells transfected with a luciferase-coupled *iNOS* reporter gene. *n* = 3; *p < 0.05. **d** Effects of lactate on cytosolic NAD(P)H levels as measured by intrinsic fluorescence (*blue*). Mitochondria are labeled with Mitotracker (*red*). *Lower row images* are enlarged views of areas defined by *rectangle in upper row. Boxes in lower row* identify a mitochondria-rich peri-nuclear region and a mitochondria-free nuclear region in one cell. **e** Example of real-time NAD(P)H fluorescence changes recorded from these two regions during incubation with 1 mM 2-deoxyglucose (added at *arrow*). **f** Quantified results showing relative cytosolic NAD(P)H fluorescence changes induced by incubation with 1 mM 2-deoxyglucose ± 20 mM lactate, glucose-free medium ± 20 mM lactate, 20 mM lactate, 200 µm cobalt chloride, or 1 µm antimycin A. *n* = 5; *p < 0.01, ^#^ *p* < 0.01 v. control. *Error bars* show s.e.m

We next asked whether the metabolic influences on the inflammatory responses are blocked when changes in the NADH:NAD^+^ ratio are negated. Lactate added to culture medium passes readily into cultured cells^33^ and drives the NADH:NAD^+^ equilibrium toward NADH (Fig. 2a), thus providing a means of countering the effect of glycolytic inhibition on the cytosolic NADH:NAD^+^ ratio. The addition of sodium lactate (20 mM, pH 7.4) reversed the effects of glycolytic inhibition on LPS-induced iNOS expression in the cell cultures (Fig. 3a, b). Evaluation of iNOS gene transcription in cells transfected with an iNOS luciferase reporter gene confirmed an effect at the level of iNOS gene transcription (Fig. 3c).

The lactate:pyruvate ratio cannot be reliably measured in cells treated with lactate, so for these studies we instead evaluated changes in cytosolic NADH by measuring changes in intrinsic NAD(P)H fluorescence. This approach does not distinguish NADH from NADPH, but cellular NADPH levels are insensitive to acute changes in glycolysis or lactate^34, 35^. Signal from mitochondrial NAD(P)H was avoided by sampling only from nuclei, which do not contain mitochondria (Fig. 3d). 2DG reduced the cytosolic NAD(P)H signal, in agreement with the lactate:pyruvate ratio determinations; and as expected 2DG had no effect on the mitochondrial NAD(P)H fluorescence (Fig. 3e). Glucose-free medium had comparable effects (Fig. 3f). The effects of both 2DG and glucose-free medium on NAD(P)H levels were reversed by sodium lactate (20 mM, pH 7.4; Fig. 3f). Although lactate did not produce a significant increase in NAD(P)H signal when administered in the absence of 2DG (Fig. 3f), the mitochondrial inhibitors cobalt and antimycin likewise failed to increase the NAD(P)H signal. NADH fluorescence measurements may be less sensitive to increases than to decreases in NADH because increased NADH is disproportionately protein-unbound, and NADH that is not protein bound has weaker intrinsic fluorescence^36^.

### 2DG suppresses NF-κB activated pro-inflammatory genes

**Fig. 4:**
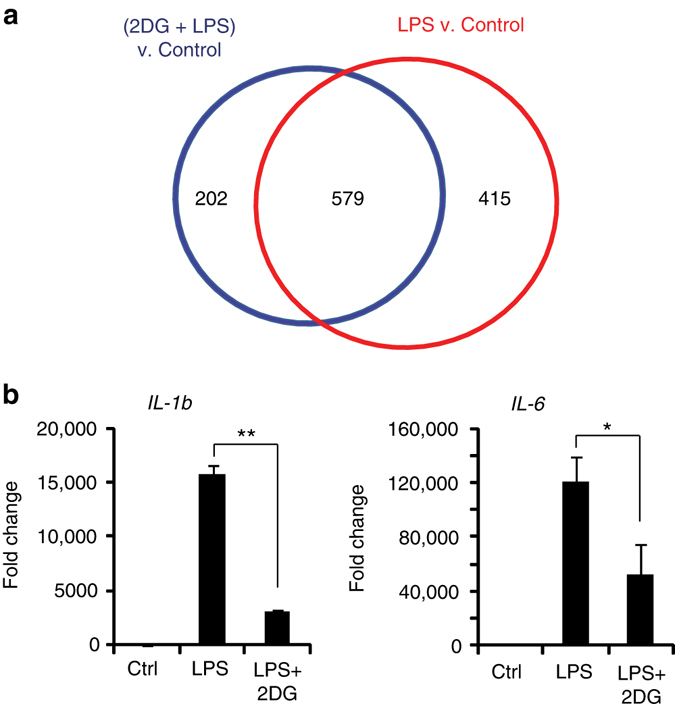
LPS and 2-deoxyglucose influence NF-κB—mediated gene transcription. **a** Microarray analysis identified 994 genes differentially regulated by LPS (*red*) and 781 genes by (2DG + LPS) (*blue*), relative to control conditions. 579 of LPS-response genes were affected by co-incubation with 2DG. **b** RT-PCR measures of NF-κB–driven pro-inflammatory cytokines confirmed that LPS-induced induction was attenuated by 2DG. *IL-1b*, interleukin-1beta; *IL-6*, interleukin-6. *n* ≥ 3; *p < 0.05, **p < 0.01. *Error bars* show s.e.m

To evaluate the broader effects of 2DG on LPS-induced gene expression, we prepared microarrays from RAW264.7 cells after incubation under control, LPS, or LPS + 2DG conditions. Expression levels of 994 genes were significantly affected by LPS and 781 by LPS + 2DG, with 579 genes responding to both conditions (Fig. 4a). An analysis of transcription factor binding sites on these genes showed that conserved NF-κB family binding sites (Rel and RelA/p65) were over-represented in the LPS + 2DG condition (Supplementary Table 1; Supplementary Data 1 and 2), as would be expected given the dominant role of NF-κB in pro-inflammatory transcriptional responses. RT-PCR quantifications of specific NF-κB—driven pro-inflammatory gene transcripts, *IL1B*, *IL6*, and *iNOS*, confirmed that they were upregulated by LPS, and that 2DG suppressed their upregulation (Figs. 1d, 4b).

### The anti-inflammatory effect of 2DG is mediated by CtBP

**Fig. 5:**
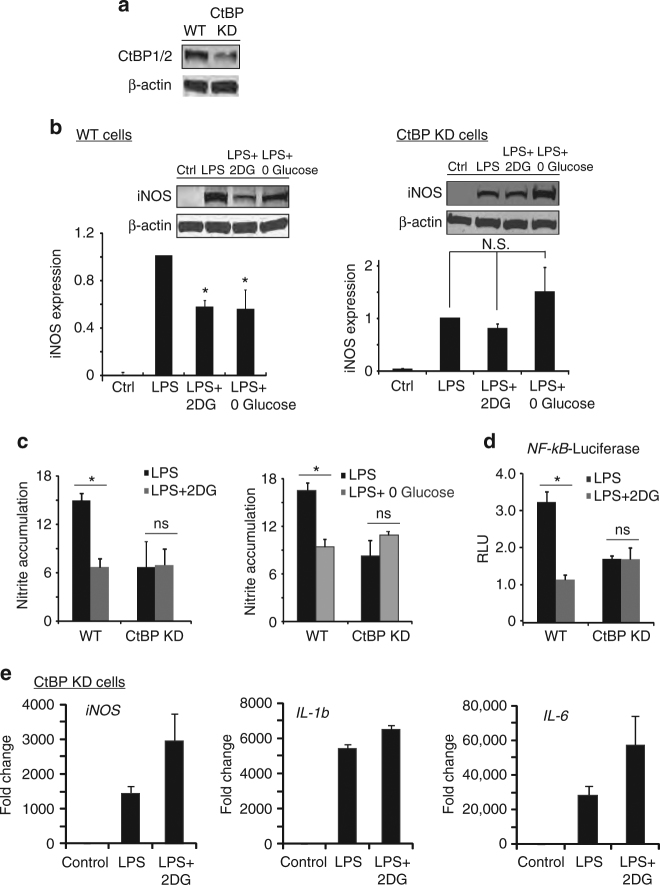
Knockdown of CtBP eliminates the effects of 2DG and glucose-free medium. **a** Representative western blot showing reduced expression of CtBP1/2 protein in RAW264.7 cells transfected with shRNA targeting CtBP1 and CtBP2 (CtBP KD). Full length immunoblots are shown in Supplementary Fig. 3. **b**, **c** shRNA knockdown of CtBP1/2 negates the effect of both 2DG and glucose-free medium on LPS-induced iNOS expression and nitric oxide production. Results for wild-type (WT) cells were normalized to control (no LPS) WT cells, and results for CtBP knockdown cells (CtBP KD) were normalized to control CtBP KD cells. *n* = 4; *p < 0.05; ns, not significant. *Error bars* show S.E.M. **d** Knockdown of CtBP1/2 negates the effects of 2DG on LPS-induced NF-κB reporter gene activation. *n* ≥ 3; *p < 0.05; ns, not significant. *Error bars* show s.e.m. **e** 2DG did not suppress LPS-induced transcription of *iNOS*, *Il-1b*, or *IL-6* in the CtBP KD cells. *n* = 3. (Compare to Figs. 1d, 4b)

We next evaluated whether the NAD(H)-sensitive transcriptional co-repressor, CtBP, is involved in the process by which energy metabolism influences inflammation. We generated a stable CtBP knockdown RAW264.7 cell line using lentivirus that expressed shRNA targeting a sequence that, in mice, is present only in *CtBP1* and *CtBP2*. The CtBP knockdown cell line displayed a 98.9 ± 0.2 % reduction in *CtBP1* and 80.8 ± 4% reduction in *CtBP2* transcripts, and a 64 ± 10% reduction in CtBP1/2 protein expression (Fig. 5a). Like wild-type cells, CtBP knockdown cells responded to LPS with increased iNOS expression and NO production (though to a lesser extent); however, unlike wild-type cells, CtBP knockdown cells showed no suppression of this response when treated with 2DG or glucose-free medium (Fig. 5b, c). Similarly, the effect of 2DG on LPS-induced NF-κB transcriptional activity was not observed in the CtBP knockdown cells (Fig. 5d), nor was the effect of 2DG on LPS-induced *iNOS*, *IL-1b*, and *IL-6* gene expression (Fig. 5e; compare to Figs. 1d, 4b).

**Fig. 6:**
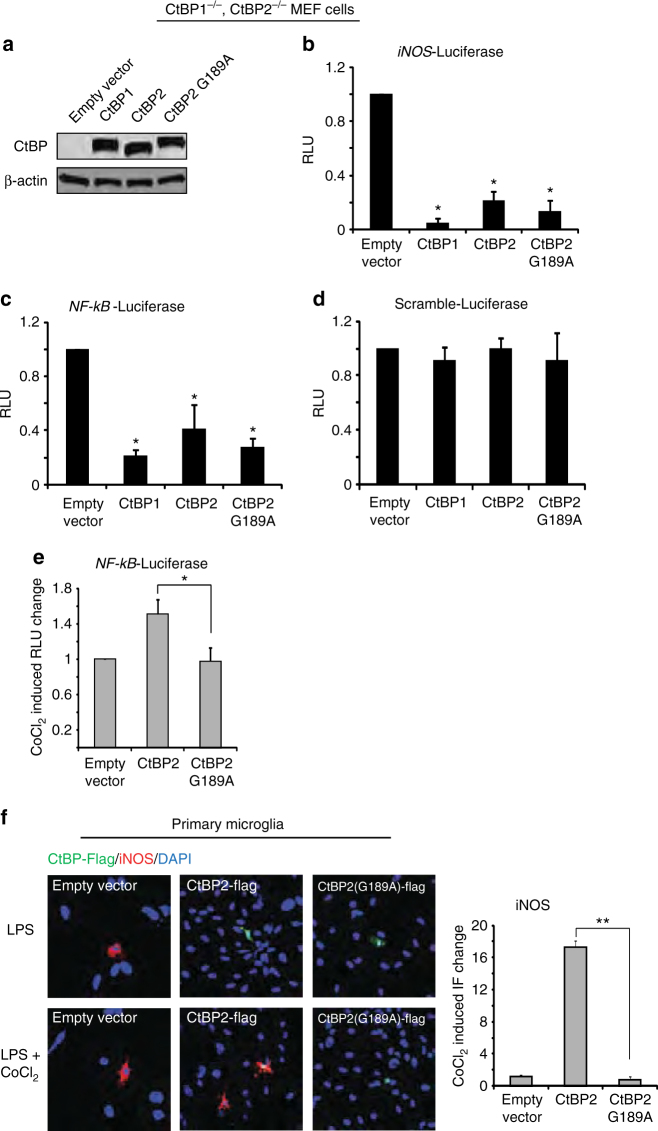
The NAD(H) binding site on CtBP is required for its effect on inflammatory responses. **a** Transfection with CtBP1, CtBP2, and G1892 CtBP2 produced comparable expression levels in CtBP1^−/−^/CtBP2^−/−^ MEF cells. Full length immunoblots are shown in Supplementary Fig. 3. **b**−**d** MEF cells were transfected with WT CtBP1, CtBP2 and G189A CtBP2, and additionally transfected with p65 to induce NF-κB activation. All 3 CtBP constructs suppress *iNOS* and *NF-κB* reporter gene transcriptional activity, and have no effect on a scrambled-sequence driven luciferase reporter gene. *n* = 3; *p < 0.05 v. empty vector. **e** The mitochondrial inhibitor CoCl_2_ increased *NF-κB* reporter gene activity in cells expressing WT CtBP2 but not in cells expressing G189A CtBP2. Results are normalized to the increase produced by CoCl_2_ in the cells transfected with empty vector alone. *n* = 4; *p < 0.05. **f** Immunostaining in primary microglia shows LPS-induced iNOS expression is potentiated by CoCl_2_ in cells transfected with WT CtBP2, but not G189A CtBP. Larger nuclei in the images belong to the astrocyte feeder layer. Results are normalized to the increase produced by 200 µm CoCl_2_ in the empty vector—transfected cells. *n* = 4; **p < 0.01. *Error bars* show s.e.m

If transcriptional effects of cytoplasmic NADH:NAD^+^ ratio are mediated by CtBP, then they should be diminished in cells expressing an NAD(H)—insensitive CtBP. The G189A mutation in CtBP2 disrupts its binding to NAD(H) while preserving its co-repressor activity^27^. We therefore determined whether the G189A mutation would negate the effect of NADH elevations on NF-κB–mediated transcriptional responses. Studies were performed in CtBP1^−/−^/CtBP2^−/−^ MEF cells in order to eliminate effects of endogenous CtBP1/2 (Fig. 6a), and because the shRNA expressed by the stable CtBP knockdown RAW264.7 cell line precludes expression of the mutant CtBP in those cells. The MEF cells were transfected with either wild-type (WT) CtBP1, WT CtBP2, or mutant G189A CtBP2 (Fig. 6a). Since the MEF cells responded only weakly to LPS (not shown), an NF-κB transcriptional response was induced by co-transfection with the p65 subunit of NF-κB. All three of the CtBP constructs suppressed NF-κB transcriptional activity, consistent with the potent co-repressor activity of CtBP (Fig. 6b–d). Treatment with 2DG produced no further reduction in this already suppressed NF-κB activity; however, treatment with CoCl_2_, which elevates the NADH:NAD^+^ ratio, increased NF-κB activity in cells expressing WT CtBP2, but not in cells expressing G189A mutant CtBP2 (Fig. 6e). This pattern was also observed in primary microglia:CoCl_2_ augmented LPS-induced iNOS expression in microglia overexpressing wild-type CtBP, but not in microglia overexpressing G189A CtBP2 (Fig. 6f).

Given that CtBP overexpression suppresses NF-κB transcriptional activity (Fig. 6c, d) we also evaluated the possibility that 2DG effects on LPS-induced responses might be caused by upregulated CtBP expression, independent of the CtBP responses to cytosolic NADH:NAD^+^ ratio. However, measures of CtBP mRNA levels in primary microglia showed that 2DG did not increase expression of either CtBP1 or CtBP2, with or without LPS co-treatment (Supplemantary Fig. 1a, b).

### Peptide inhibition of CtBP dimerization

**Fig. 7:**
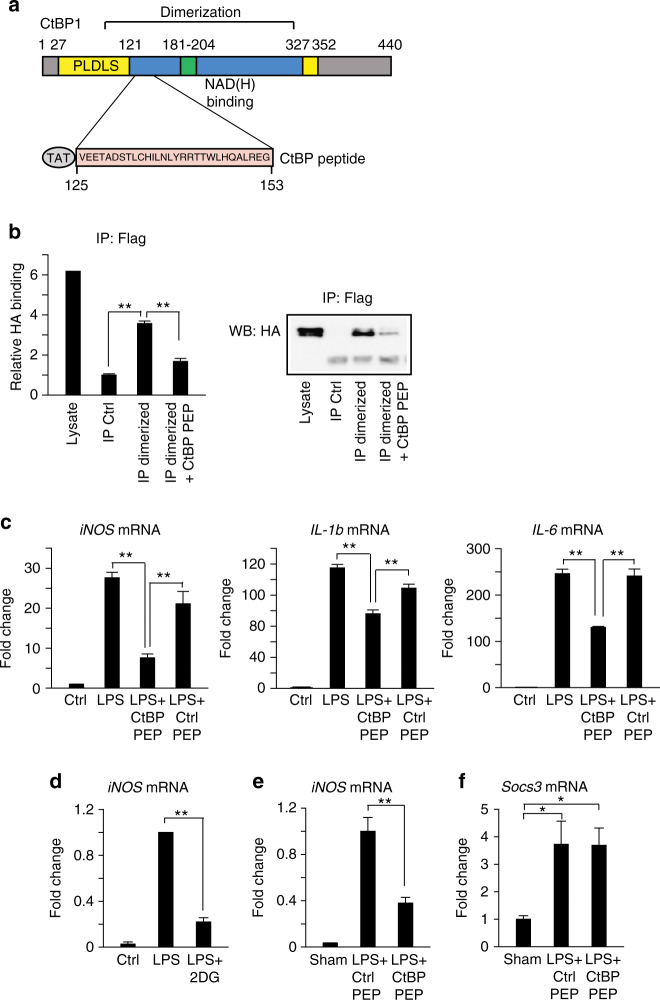
Direct inhibition of CtBP dimerization blocks LPS-induced pro-inflammatory gene expression. **a** Schematic of CtBP1 protein showing functional domains and alignment with the CtBP peptide used to block CtBP dimerization. PLDLS indicates substrate binding domain. The CtBP blocking peptide includes an N-terminal TAT sequence for cellular internalization. **b** Immunoprecipitation assay showing ability of CtBP peptide (50 µm) to block dimerization of tagged CtBP proteins incubated with 10 mM lactate + 10 µm NADH. Full length immunoblots are shown in Supplementary Fig. 3. Immunoprecipitation was performed with anti-Flag antibody, and CtBP1-Flag/CtBP1-HA heterodimers were detected by western blots using anti-HA antibody. Lysate control lane = 5% of input used in immunoprecipitated samples. *n* = 4. **p < 0.01. **c** CtBP peptide (5 µm) blocks LPS-induced mRNA expression of pro-inflammatory genes (iNOS, IL-1b and IL-6) in cultured primary microglia. Ctrl PEP = control peptide. *n* ≥ 3; **p < 0.01. **d** 2DG blocks LPS-induced iNOS gene expression in microglia isolated from mice after LPS (8 µg) injection into striatum. *n* = 3–5 per group. **p < 0.01. **e**, **f** CtBP peptide blocks LPS-induced iNOS expression but not Socs3 expression in brain microglia. *n* = 4 per group. **p < 0.01. *Error bars* show s.e.m

To further evaluate the mechanism by which CtBP regulates inflammatory responses, we generated a synthetic peptide that blocks CtBP dimeriziation. Examination of the crystal structure of a rat CtBP dimer complexed with NAD(H)^37^ suggested that a peptide near the center of the dimerization region, if stably folded, could bind to the CtBP monomer and interfere with dimer formation. This dimerization region contains two alpha helical domains that are structurally conserved in CtBP family members, and contains four residues that, when mutated, prevent dimerization of drosophila CtBP^22^. A peptide spanning this region, corresponding to amino acids 114−142 of the long form of mouse CtBP1 (Fig. 7a), and a control peptide lacking any known effects on cell function were generated and fused to N-terminal Tat sequences (CPC Scientific (Sunnyvale, CA). The CtBP peptide sequence was **GRKKRRQRRRC** VEETADSTLCHILNLYRRTTWLHQALREG (with the Tat sequence underlined), and the control peptide sequence was **GRKKRRQRRRC** CSFNSYELGSLCYGRKKRRQRR. To determine if the CtBP peptide could block CtBP dimerization in vitro, we co-expressed CtBP1-HA and CtBP1-Flag tagged constructs in COS7 cells and performed immunoprecipitation with antibody to Flag. Very little HA-tagged CtBP was immunoprecipitated with anti-Flag antibody in untreated control samples (Fig. 7b), suggesting that the majority of the CtBP-tagged protein is monomeric. In contrast, in vitro treatment of lysate with 10 mM lactate plus 10 µm NADH for 20 min prior to immunoprecipitation (to force an increase in the NADH:NAD^+^ ratio in the lysates) greatly increased the amount of CtBP1-HA associated with CtBP1-Flag. This association was disrupted by co-incubation with 50 µm of the CtBP peptide (Fig. 7b).

We next tested whether the CtBP peptide could inhibit LPS-induced inflammatory gene expression, as observed in the 2DG—induced ketogenic state. Primary microglia were treated with LPS, alone or in combination with 5 µm CtBP peptide or control peptide. The CtBP peptide reduced LPS-induced iNOS, IL-1b, and IL-6 transcript expression, while the control peptide had no effect (Fig. 7c). We then evaluated whether the CtBP peptide could block inflammatory gene expression in vivo. In initial experiments, we treated mice intraperitoneally with 2DG (100 mg/kg) 10 min prior to stereotactic injection of 8 µg LPS into the left striatum. Three hours of post-surgery, brains were removed and microglia were isolated from the striatum for gene expression studies. These studies confirmed that LPS robustly induces iNOS expression in microglia in vivo, and that this effect is attenuated by 2DG (Fig. 7d). We then co-injected LPS (8 µg) along with the CtBP peptide or control peptide into the striatum 3 h prior to microglial isolation. The LPS-induced increase in iNOS gene expression was reduced by the CtBP peptide, but not by the control peptide. In contrast, LPS-induced expression of the M2-type anti-inflammatory gene, Socs3 (Suppressor of cytokine signaling 3)^38^, was unaffected by CtBP peptide, demonstrating that the peptide does not exert a global or nonspecific effect on transcription (Fig. 7e, f).

### p300 binding to CtBP and pro-inflammatory gene promoters

**Fig. 8:**
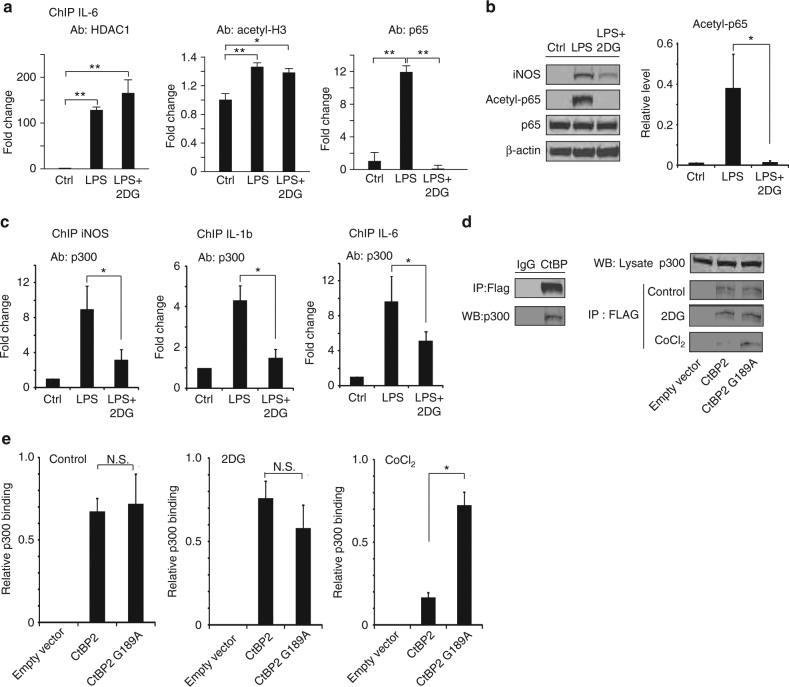
CtBP effects on p300 and NF-κB acetylation. **a** Chromatin immunoprecipitation (ChIP) with antibody to HDAC1, acetyl H3, and p65 was performed in RAW264.7 cells to evaluate binding to the IL-6 promoter regions. LPS increased all three signals, but only the effect of p65 binding was reversed by 2DG (conditions as in Fig. 1c); *n* = 3, *p < 0.05. **b** Western blots show LPS-induced p65 acetylation is suppressed by 2DG (conditions as in Fig. 1c); *n* = 3, *p < 0.05. Full length immunoblots are shown in Supplementary Fig. 3. **c** ChIP was performed to evaluate p300 binding to NF-κB p65 binding sites on promoter regions of pro-inflammatory cytokines. p300 binding was increased by LPS, and this effect was attenuated by 2DG (conditions as in Fig. 1c); *n* = 3, *p < 0.05. **d** HEK293 cells were transfected with FLAG-tagged WT CtBP2 or G189A CtBP2. Immunoprecipitation using antibody to FLAG recovered p300 protein, while antibody to IgG, used as a negative control, did not. Immunoprecipitates from transfected cells treated with 2DG or CoCl_2_ for 30 min showed reduced p300 binding to WT CtBP2, but not G189A CtBP2, in cells treated with CoCl_2_. **e** Quantified results of the immunoprecipitation studies. Results were normalized to p300 in the lysate input. *n* = 3; *p < 0.05. *Error bars* show s.e.m

One way that CtBP regulates gene transcription is through interactions with the histone acetyltransferase HDAC1^19^. We therefore performed chromatin immunoprecipitation (ChIP) targeting the IL-6 promoter to evaluate the effects of ketogenic state on HDAC1 binding and histone H3 acetylation. Both HDAC1 binding and H3 acetylation were increased in cells treated with LPS, but neither was reversed by 2DG (Fig. 8a). A second way that CtBP regulates gene transcription is by repressing the activity of p300^20, 39^, which acetylates and thereby promotes the activity of transcription factors such as the p65 subunit of NF-κB^25^. The ChIP studies showed that LPS-induced p65 binding to the IL-6 promotor, and that this was completely reversed in the presence of 2DG (Fig. 8a). This effect of 2DG was accompanied by parallel changes in p65 acetylation status, as assessed by western blots from cells treated with LPS or LPS + 2DG (Fig. 8b). Additional ChIP studies showed that LPS-induced p300 binding to the IL-6, IL-1b, and iNOS promoter regions, and that 2DG markedly attenuated each of these effects (Fig. 8c). These results parallel the effects of LPS and 2DG on iNOS, IL-1b, and IL-6 mRNA and protein expression (Figs. 1, 4). We then performed co-immunoprecipitation of p300 with CtBP in cells transfected with either wild-type CtBP2 or G189A CtBP2 to confirm that p300 binding to CtBP is similarly sensitive to NADH. 2DG—induced reductions in the cytosolic NADH:NAD^+^ ratio did not increase the binding of p300 to either wild-type CtBP2 or G189A CtBP2, presumably because binding was already saturated by CtBP overexpression; however, CoCl_2_—induced elevations in the NADH:NAD^+^ ratio reduced the binding of p300 to wild-type CtBP, but not G189A CtBP2 (Fig. 8d, e).

## Discussion

CtBP has previously been shown to mediate effects of cellular metabolic state on transcriptional events contributing to cancer and epilepsy^27, 40^. Our present findings demonstrate that CtBP similarly couples metabolic state to the innate inflammatory response. We show that the pro-inflammatory gene expression and NF-κB transcriptional activity induced by LPS are coupled to glucose metabolism by the cytosolic NADH:NAD^+^ ratio, and that this coupling is attenuated by CtBP downregulation, by forced elevation of NADH, by transfection with an NADH—insensitive CtBP, and by a synthetic peptide that blocks NADH-induced CtBP dimerization. We additionally show that metabolic state influences CtBP binding to the acetyltransferase p300, p300 binding at pro-inflammatory gene promoter sites, and acetylation state of NF-κB. The observations suggest a mechanism by which the liberation of CtBP from its dimerized form by lowered cytosolic NADH levels can suppress pro-inflammatory gene transcription.

The initial observation by Zhang et al.^26^ that NADH binding promotes CtBP dimerization suggested that CtBP could regulate transcription in response to metabolic changes. Subsequent studies confirmed that CtBP mediates effects of 2-deoxyglucose, hypoxia, pyruvate, and other metabolic influences on gene expression^27, 41^. Since NAD^+^ and NADH recognize the same binding site on CtBP, changes in the concentration of either nucleotide could, in principle, regulate CtBP interactions with its binding partners. However, the relative changes in NADH caused by shifts in the cytosolic NADH:NAD^+^ ratio are several hundred-fold greater than the reciprocal changes in NAD^+^ because the cytosolic NADH:NAD^+^ ratio is normally in the range of 1:700^35^. The sensitivity of CtBP to changes in NADH thus makes it particularly responsive to changes in energy metabolism^42^. Inflammatory responses can also be modulated by NAD^+^—dependent deacetylases of the sirtuin family, notably Sirt1^43^, but unlike CtBP sirtuins are not responsive to changes in NADH concentrations, and it remains uncertain whether their activity is significantly affected by the relatively small changes in NAD^+^ concentrations that result from changes in energy metabolism^44^.

CtBP1 and CtBP2 have nearly identical amino acid sequences and share the same NAD(H) binding site. CtBP1 lacks a putative nuclear localization signal, but may enter the nucleus as heterodimer with CtBP2 or other proteins^22, 45^ and may also interact in the extra-nuclear cytosol with binding partners such as p300 and HDAC1. CtBP2^−/−^ mice are nonviable, but CtBP1^−/−^ mice are viable and fertile. Mice with combinations of CtBP1 and CtBP2 mutant alleles exhibit gene dosage-sensitive defects in several developmental processes^17^. Specific defects in immune function were not reported in these mutants, but CtBP has elsewhere been shown to modulate the role of estrogen receptors on inflammatory responses mediated by the AP-1 transcription factor, independent of metabolic changes^46^.

CtBP regulates gene transcription in multiple ways, including interactions with histone deacetylases, histone methyl transferases, E3 ligases, and the p300 acetyltransferase. We did not find changes in histone acetylation or HDAC1 binding at pro-inflammatory gene promoters in response to a ketogenic state, but did find decreased binding of p300 and decreased acetylation of p65, which is a substrate for p300^25^. Since p65 acetylation increases NF-κB transcriptional activity, these findings identify a mechanism by which metabolic effects on CtBP can modulate gene transcription. However, both monomer and dimer forms of CtBP can affect gene transcription^21–24^, and our findings do not establish that p300/CtBP binding is essential for the observed effects of CtBP on pro-inflammatory gene transcription. CtBP2 could alternatively directly associate with p65 and suppress its acetylation by a mechanism independent of dimer/monomer transition or p300 inhibition^47^. Recent studies have also identified alternative ways that glucose metabolism can influence inflammatory responses, including effects on HDAC4 protein levels, NLRP3 inflammasome formation, and the activation of RAGE receptors^48–50^.

Cell cultures were used in our studies to facilitate manipulation of CtBP expression and cellular cytosolic NADH:NAD^+^ ratios. A limitation cell cultures, and cell lines in particular, is a shift from respiratory ATP production toward more glycolytic ATP production (the Warburg effect^51^). Moreover, the glucose concentration in standard culture media is saturating for glucose uptake and metabolism. As a consequence, basal glycolytic flux is typically much higher in culture preparations than in vivo. This may explain why relatively larger shifts in the cytosolic NADH:NAD^+^ ratio were observed with glycolytic inhibition than with respiratory inhibitors. However, the anti-inflammatory effects of 2DG in the cell culture preparations were also observed in vivo, as initially reported several decades ago^6^. Importantly, these results were also mimicked, both in vitro and in vivo, by the use of a novel synthetic peptide that inhibits NADH-induced CtBP dimerization. This result agrees with the prior observation that native CtBP1 requires NADH to suppress p300 activity, whereas a mutant form of CtBP1 that cannot dimerize suppresses p300 independent of NADH^20^ It may be surprising that reduced CtBP expression did not itself lead to a super-induction of iNOS and related genes, given co-repressor actions of CtBP. However, a reduction in total CtBP does not necessarily produce a comparable reduction in nuclear monomeric CtBP.

Taken together, our findings indicate that metabolic influences that alter the cytosolic NADH:NAD^+^ ratio regulate NF-κB transcriptional activity through an NADH-dependent effect on CtBP dimerization. Conditions that reduce glycolytic flux, such as ketogenic diet and caloric restriction, can thereby suppress NF-κB activity, while conditions that increase glycolytic flux may increase it. These interactions provide a mechanism for the suppressive effects of ketogenic diet and caloric restriction on brain inflammation after brain injury. By extension, these interactions may also contribute to the pro-inflammatory states associated with diabetes mellitus and metabolic syndrome^15, 52, 53^.

## Methods

### Reagents

The MEF cell lines (*CtBP1* ^*−/−*^ */CtBP2* ^*−/−*^ and *CtBP1* ^*−/+*^ */CtBP2* ^*−/+*^) were a kind gift from Dr. A. Roopra (University of Wisconsin, Madison). HEK293 cells and the macrophage-derived RAW264.7 cell line were obtained from the ATCC. The p*NF-κB-RE*-firefly and pRL-CMV renilla luciferase reporter were obtained from Promega, and the control pTA-luciferase reporter was obtained from Panomics. Full length cDNAs of mouse *CtBP1* and *CtBP2* were cloned into pIRES-hrGFP-1a (Agilent) from adult mouse brain cDNA by reverse transcriptase PCR. The CtBP2 G189A mutant was a gift from Dr. J. Blaydes (University of Southampton, UK) and was cloned into pIRES-hrGFP-1a. The *iNOS* promoter-luciferase reporter was a gift from Dr. C. Lowenstein (University of Rochester, Addgene plasmid 19,296)^54^, the p300 plasmid was a gift from Dr. W. Sellers (Dana-Farber Cancer Institute, Addgene plasmid 10,717), the p65 plasmid was a gift from Dr. W. Greene (UCSF, Addgene plasmid 21,966), and the flag-tagged CtBP2 plasmid was a gift from Dr. A. Roopra (University of Wisconsin, Madison). GIPZ-puro vector-based lentiviral vectors containing CtBP1/2 shRNA were purchased from Open Biosystem (Thermol Scientific), and purified lentivirus transduction particles were generated by the UCSF ViraCore Laboratory. All constructs were sequence verified. All other reagents were obtained from Sigma-Aldrich except where noted.

### Surgical procedures and microglial isolation

Studies were approved by the San Francisco Veterans Affairs Medical Center animal studies committee. Male Sprague Dawley rats, age 4–6 months (Charles River Laboratories), were given intraperitoneal injections of LPS (10 mg/kg) or saline vehicle, with or without 2-deoxyglucose (2DG; 100 mg/kg)^55^. Twenty-four hours later the rats were anesthetized and perfused with saline followed by 4% formaldehyde. Coronal 40 μm cryostat sections were prepared and immunostained^56^ for CD11b (Serotec, clone OX-42, 1:100 dilution). Images were acquired using fluorescence confocal microscope and quantified as described^57^.

Stereotaxic LPS injections were performed in adult male C57BL/6 J mice that were anesthetized with 2% isoflurane. The left striatum (A 1.3, ML 1.9, DV 3.5 mm from Bregma and the cortical surface) was injected with 8 µg LPS and 5 pmole CtBP peptide or control peptide in a volume of 5 µl sterile saline over 25 min (0.2 µl/min). Three hours later the mice were transcardially perfused with saline to remove circulating macrophages. After perfusion, brains were quickly removed and the left anterior quadrant containing the infusion site (approximately 100 mg tissue) was dissected and placed in ice-cold Hank’s Balanced Salt Solution (HBSS) without Ca^2+^ and Mg^2+^. Tissue was briefly minced with a sterile razor blade and cells were dissociated using Neural Tissue Dissociation Kit (P) and Octo Dissociator (Miltenyi Biotec, San Diego, CA). Microglia were isolated using CD11b MicroBeads (Miltenyi Biotec). Accuracy of cell separation was validated by expression profiling of cellular fractions using quantitative PCR primers specific to microglia (Iba1 forward: 5′-GAAGCGAATGCTGGAGAAAC, reverse: 5′-GACCAGTTGGCCTCTTGTGT), neurons (NeuN forward: 5′-GGAACAGTCTATGGGCCTGA, reverse: 5′-ACAAGAGAGTGGTGGGAACG) and astrocytes (GFAP forward: 5′-AGAAAGGTTGAATCGCTGGG, reverse: 5′-CGGCGATAGTCGTTAGCTTC) (Supplementary Fig. 2).

### Hippocampal organotypic slice cultures

Slice cultures were prepared as described^58^, with minor modifications. Brains were removed from 4-day-old male mice C57BL/6 mice, and 350 µm sagittal sections were prepared with a vibratome in ice-cold dissecting media. The hippocampi were placed on Millicell inserts in 6 well culture plates, and maintained for 2 weeks at 37 ˚C in 5% CO_2_/ 95% air incubator. Experiments were initiated by adding 10 μg/ml LPS ± 1 mM 2DG. After 24 h the slices were fixed with 4% formaldehyde. Slices were immunostained using rabbit polyclonal antibody to Iba1 (Wako #019-19,741; 1:200 dilution) and rabbit polyclonal antibody to iNOS (Millipore, # 06-573; 1:250 dilution).

### Cell cultures

Mouse primary microglia cells were isolated and cultured as described^56^ and used after 10 days in vitro. Cell lines were seeded at a density of 1 × 10^4^ cells/well in 24-well plates 1 day prior to the experiments. For protein expression and luciferase assays, MEF cells, RAW264.7 cells and HEK293 cells were seeded in 12-well or 24-well plates overnight, and transfected with plasmids using Lipofectamine2000 (Invitrogen) when they reached 80% confluence. A stable CtBP1/2 knockdown cell line was generated from RAW264.7 cells by incubating the cells with CtBP1/2 shRNA lentiviral stocks for 3 h. Puromycin was added after 48 h, and infected cells were selected over 10 passages. Stable clones were pooled for propagation and experiments.

Studies were initiated by washing cells into serum-free Dulbecco’s minimal essential medium containing 6 mM glutamine and 2 mM glucose (or no glucose, where indicated). Lipopolysaccharide (LPS), lactate, and metabolic inhibitors were added to this medium from concentrated stocks that were pre-adjusted to pH 7.4, and the cultures were replaced in a 37 °C, 5% CO_2_ incubator. The final LPS concentration was 10 ng/ml in the cell cultures. All experiments were repeated at least three times, using triplicate wells in each experiment.

### ATP measurements

Cells were washed with ice-cold phosphate-buffered saline and extracted with ice-cold 0.5% trichloroacetic acid. The cell lysate pH was adjusted to 7.8 with 1 M Tris-base. ATP concentration was determined by a luciferase-linked method (Promega Enlighten ATP kit) using ATP standards. Values were normalized to protein concentrations as determined by the Bradford assay (Bio-Rad) in sister culture wells.

### Lactate and pyruvate assays

RAW264.7 cells were rinsed with ice-cold phosphate-buffered saline, lysed in 0.2 N NaOH, and aliquots were taken for protein assay. Lactate and pyruvate were measured in pH-neutralized lysates by enzyme-linked assays as described^12^ using standards treated identically.

### NADH imaging

Real-time changes in cellular (cytoplasmic) NADH content were estimated by measuring endogenous NADH fluorescence with 360 nm excitation and > 410 nm emission^59^. Fluorescence images were acquired at 1 min intervals after 5 min of baseline recording. Regions of interest were selected from the nucleus to eliminate signal from mitochondria, and cytochalasin D (1 µm) was included to prevent cell movement. Images acquired from each cell were used to calculate change in fluorescence intensity / average of pre-treatment fluorescence intensity (Δ*F*/*F*_o_). In a subset of experiments cells were pre-incubated with 200 nm Mitotracker Red-FM (Molecular Probes) to verify that the NADH fluorescence measurements were not contaminated by mitochondrial NADH fluorescence. Mitochondrial and NADH signals were imaged by interleaving NADH fluorescence excitation and Mitotraker (excitation 550 nm; emission > 610 nm).

### Nitric oxide measurements

Production of nitric oxide was assessed by analyzing nitrite concentrations in the culture medium collected after 24 h incubation under the designated conditions. Aliquots of culture medium or nitrite standards prepared in culture medium were mixed with equal volumes of Griess reagent and light absorbance was measured at 540 nm^56^. Values were normalized to the protein content of each culture well.

### Luciferase reporter gene assays

Cells were transfected with 300 ng of *5xNF-κB* or *iNOS* firefly luciferase constructs using Lipofectamine2000 (Life Technologies), and co-transfected with 100 ng of GL3-Renilla luciferase construct (Promega) as an internal control of transfection efficiency. In some experiments cells were also transfected with a CtBP constructs (200 ng) or CtBP empty control vector. Renilla luciferase and firefly luciferase activity were measured in cell lysates using a dual luciferase assay kit (Promega) on a Modulus Microplate Reader (Turner Biosystems). Reporter luciferase activity was normalized to Renilla luciferase activity in each assay.

### Microarray and differential expression analysis

Cells were collected after 24 h incubation under the designated conditions. RNA was extracted and arrayed in triplicates using Mouse OneArray chip (Phalanx Biotech). Data analysis was performed using Bioconductor packages in R (www.bioconductor.org). Raw reads from microarrays were first normalized across the samples using the ‘vsn’ package from Bioconductor^60^. The normalized expression levels of the samples (with technical and experimental duplicates) were then fitted with a mixed linear model using the ‘limma’ package from Bioconductor^61^. Differential expression between pairs of treatment factors were also calculated using the ‘limma’ package, and the resulting *p*-values were adjusted for multiple tests using the Benjamin-Hochberg procedure. A Venn diagram was also generated to visualize the overlap of differentially expressed genes between pairs of factors among three factors, using R codes adapted from the ‘limma’ package. Shared transcription factor binding sites were identified by blast differentially expressed genes in the oPOSSUM server (http://opossum.cisreg.ca/oPOSSUM3/) with a filter setting of *Z*-score above 15.

### Quantitative real-time PCR analysis

RNA was extracted from freshly harvested cells (Qiagen), treated with DNAse I (Promega) and first-strand cDNA was synthesized from 2 μg total RNA with Oligo (dT) primers (Life Technologies). Real-time PCR with SYBR green detection (Applied Biosystems) was performed as described previously^62^ using an ABI 7900HT FAST Real-Time PCR System. Dilution series and standard curves of β-actin were amplified on each plate for all experiments. Transcript levels of all genes from each sample were normalized to its ß-Actin mRNA level using the 2^−ΔΔCT^ method^62^.

### Co-immunoprecipitation and western blots

Cells were lysed with M-PER reagent (Pierce) supplemented with benzonase nuclease (Novagen) and protease inhibitors (Roche). Soluble lysate was centrifuged for 10 min at 4 °C and the supernatant was incubated with anti-Flag M2 agarose (Sigma) overnight. The M2 agarose was washed five times with 0.25% Triton X-100 / phosphate-buffered saline and three times with 0.5% Triton X-100/phosphate-buffered saline. Bound protein was eluted using 1X Sample Loading Buffer (Invitrogen) and heated to 100 °C for 5 min. Lysate and eluate were resolved on 10% SDS-PAGE gels, and transferred to PVDF membranes (Millipore)^62^. The blots were probed with mouse polyclonal anti iNOS (Upstate, #06-573; 1:1000), rabbit polyclonal anti ß-actin (Sigma-Aldrich, #A2066; 1:1000), and mouse monoclonal anti-CtBP (AbNova, # H1487-MO1, 1:1000).

### Chromatin immunoprecipitation

Cells were treated with 1% formaldehyde for 10 min at 37 °C and rinsed twice with ice-cold phosphate-buffered saline supplemented with phenylmethanesulfonyl fluoride and protease inhibitors (Roche). Cells were then lysed, sonicated on ice, and centrifuged. Aliquots of the supernatants were heated to reverse crosslinks and recover genomic DNA for an input control. A ChIP assay kit (Millipore) was used with mouse antibody to CtBP (AbNova, # H1487-MO1, 1:250 dilution) and rabbit antibodies to p300, HDAC1, and p65 (Santa Cruz Biotechnology; sc-585, sc-7872, sc-372) or acetyl H3 (Cell Signaling, #8173), each at 1:250 dilutions, and with mouse and rabbit IgG controls (Cell Signaling). Precipitated material was eluted by two 15 min incubations at room temperature with 250 μl of 1% SDS / 0.1 M NaHCO_3_. Chromatin was crosslink-reversed and submitted to RNAse and proteinase K digestion. DNA was extracted by phenol-chloroform and the DNA from both the ChIP and the input controls were analyzed by quantitative real-time PCR analysis. Primers targeting the iNOS, Il-1b, and Il6 promoter regions were as published^63, 64^.

### Peptide design and synthesis

The crystal structure of a rat CtBP dimer complexed with NAD(H) (PDB 1HKU)^37^ using Accelrys DS Viewer 1.7 suggested that a peptide near the center of the dimerization region, if stably folded, might interfere with dimer formation. This dimerization region contains two alpha helical domains that are structurally conserved in CtBP family members and also contains residues that, when mutated, prevent dimerization of drosophila CtBP^22^. A corresponding CtBP peptide along with a control peptide lacking any known cell function were generated and fused to an N-terminal Tat sequence by CPC Scientific (Sunnyvale, CA). The CtBP peptide sequence was **GRKKRRQRRRC** VEETADSTLCHILNLYRRTTWLHQALREG (with the Tat sequence underlined), and the control peptide sequence was **GRKKRRQRRRC** CSFNSYELGSLCYGRKKRRQRR.

### CtBP dimerization assay

CtBP1-HA and CtBP1-Flag expression constructs were generated by PCR amplification of full length CtBP1 coding sequence from mouse brain-derived cDNA, followed by cloning in-frame into pSelect-CHA-zeo (Invivogen, San Diego, CA) and pCMV-(DYKDDDDK)-C (Clontech, Mountain View, CA) vectors, respectively. Clone identity and integrity were verified by sequence analysis. COS7 cells were grown to 75% confluence in 6-well plates in Dulbecco’s minimal essential medium containing fetal bovine serum and 230 µm sodium pyruvate. Five micrograms of each plasmid per plate was transfected using Lipofectamine2000 (Life Technologies, Inc., Grand Island, NY). 24 h post transfection, cells were washed once in 1X PBS and lysed in NP-40 buffer (20 mM Tris-HCl pH 8.0, 137 mM NaCl, 10% glycerol, 1% Nonidet P-40, 2 mM EDTA) containing Complete Mini protease inhibitor cocktail (Roche, Indianapolis, IN). Lysates were cleared of cellular debris by brief centrifugation and concentration determined by Pierce BCA Protein Assay (Thermo Scientific, Rockford, IL). Forty micrograms of protein from each sample lysate was incubated at 37 °C for 20 min either with or without the addition of 50 µm CtBP peptide. In some studies incubation solution also contained 10 mM sodium lactate and 10 µm NADH to promote CtBP dimerization. Co-immunoprecipitation was performed by overnight incubation of samples with 2 µg mouse monoclonal ANTI-FLAG M2 antibody (Sigma-Aldrich, # F3165), 3 h incubation with 40 µl prewashed protein A/G-Sepharose beads (Biovision Inc., Milpitas, CA) and serially washed with NP-40 buffer per manufacturer’s instructions. Bound protein was eluted from Sepharose beads by addition of NuPAGE LDS Sample buffer (Life Technologies, Inc.) and analyzed by standard SDS-PAGE gel electrophoresis using polyclonal rabbit anti-HA antibody (Thermo Scientific, # PA1-985, 1:500 dilution).

### Statistical analyses

In all cell culture or slice culture studies the ‘*n*’ values denote the number of independent experiments, each using neurons prepared from different mice. Each independent experiment contained triplicate culture wells. Data other than the microarray results are expressed as means ± SEM and assessed using one-way ANOVA followed by either the Tukey–Kramer test where multiple groups are compared against one another, or Dunnett’s test where multiple groups are compared against a common control group. All data analyses were performed while blinded to the treatment conditions.

### Data availability

The microarray data that support the findings of this study are available in FigShare with the identifier https://doi.org/10.6084/m9.figshare.c.3828235.v1. The authors declare that all other relevant data supporting the findings of the study are available in this article and its Supplementary Information files, or from the corresponding author upon request.

## Electronic supplementary material

Supplementary information

Supplementary Data 1

Supplementary Data 2
