# Supplementary material for: Bioenergetic state regulates innate inflammatory responses through the transcriptional co-repressor CtBP
Source: Nat Commun. 2017 Sep 22;8:624. doi: 10.1038/s41467-017-00707-0 (PMC5608947; doi:10.1038/s41467-017-00707-0)

Description of Supplementary Files

File Name: Supplementary Information

Description: Supplementary figures, supplementary table

File Name: Supplementary Data 1

Description: Microarray data template

File Name: Supplementary Data 2

Description: Microarray data

Supplementary Table

Transcription Factor	JASPAR ID	Family	Target Gene hits	Target Transcription factor binding site hits	Z-score
NFkB1	MA0105.1	Rel	126	214	21.4
NF-kappaB	MA0061.1	Rel	257	615	20.3
Rel	MA0101.1	Rel	311	922	18.4
RelA	MA0107.1	Rel	224	449	18.2
Sp1	MA0079.2	Beta Beta Alpha-zinc finger	351	1971	16.0
Klf4	MA0039.2	Beta Beta Alpha-zinc finger	393	2380	15.64
MZF1_5-13	MA0057.1	Beta Beta Alpha-zinc finger	394	2148	15.2

Analysis of transcription factor binding sites in the 579 genes found to be affected by both the LPS and LPS+2DG treatments with Z-score above 15. Z-score indicates the degree to which the number of transcription factor binding site (TFBS) per gene in this gene set deviates from the expected number in a random set of 579 genes from the mouse genome database. NF-κB –family binding sites (Rel and RelA) were the most over-represented transcription factor binding sites in this gene set.

Supplemental Figure 1

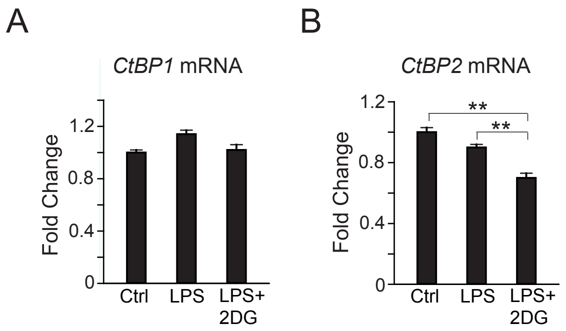

2DG and LPS do not increase CtBP expression. (A) CtBP1 and (B) CtBP2 transcript levels in primary microglia after incubation for 24 hours with LPS (10 ng / ml) and 2DG (1 mM). n = 5; **$p < 0.01$. Error bars show s.e.m.

Supplemental Figure 2

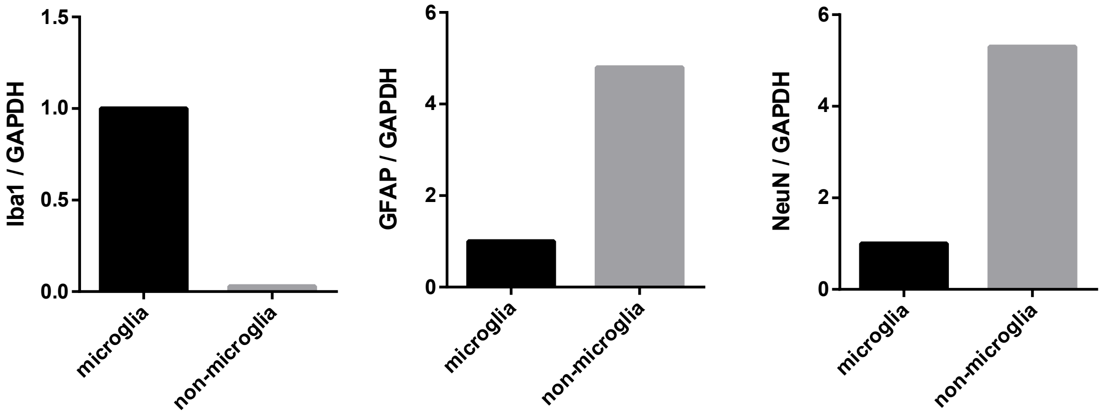

Expression profiling of microglia isolated from mouse brain. RT-PCR was used to measure expression of the microglial marker Iba1, the astrocyte marker GFAP, and the neuronal marker NeuN in the putative microglia and non-microglia fractions. Gene expression in each fraction was normalized to GAPDH and compared to the microglial fraction.

Supplemental Figure 3. Uncropped western blot images

iNOS

1. Control
2. LPS
3. LPS+2DG
4. 2DG

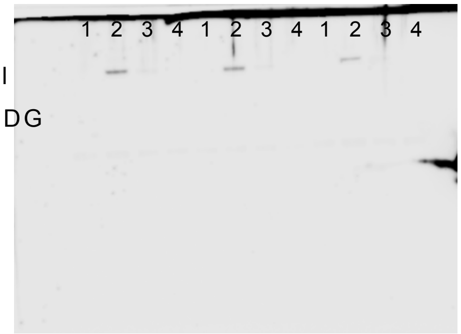

β-Actin

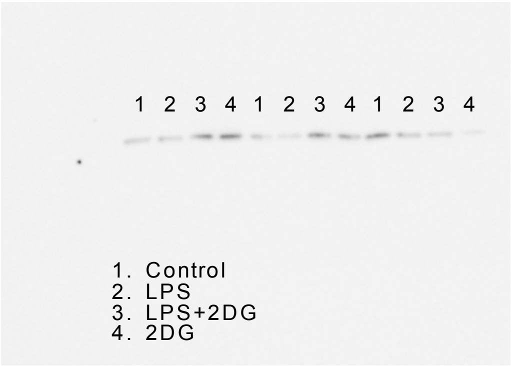

Full length immumoblots from Fig. 1C

Control LPS 2DG+LPS

iNOS

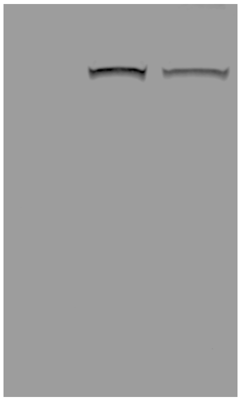

Control LPS 2DG+LPS

β-Actin

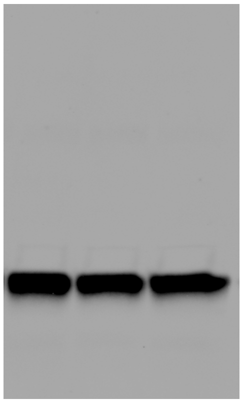

Full lenght immunoblots from Fig. 1D

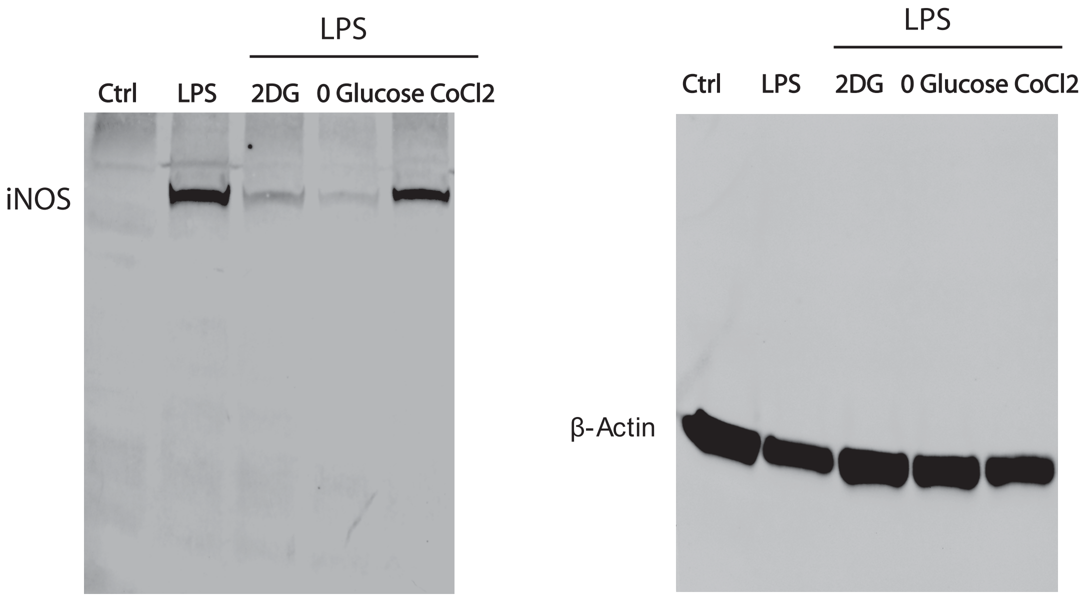

Full length immunoblots from Fig. 2C

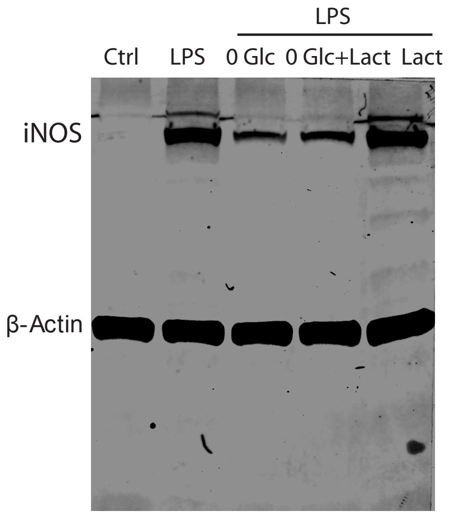

Full length immunoblot from Fig. 3A

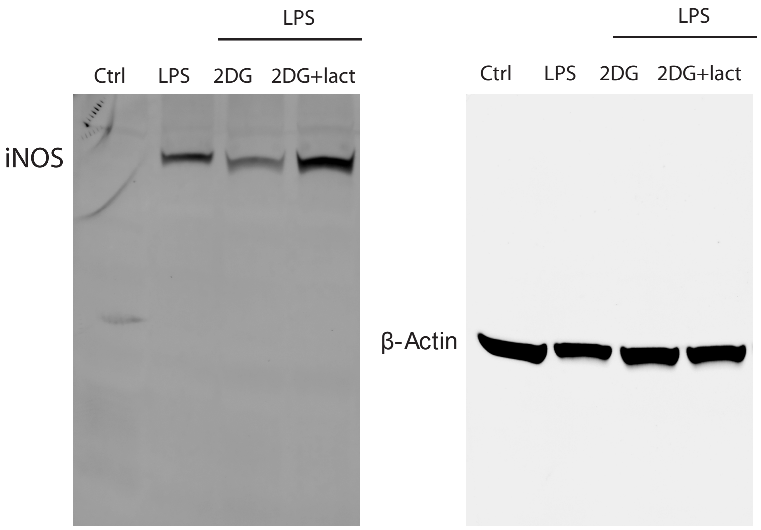

Full length immunoblots from Fig. 3B

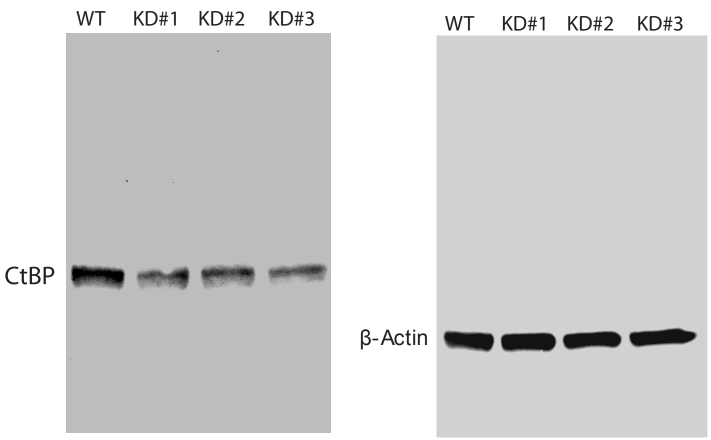

Full length immunoblots from Fig. 5A

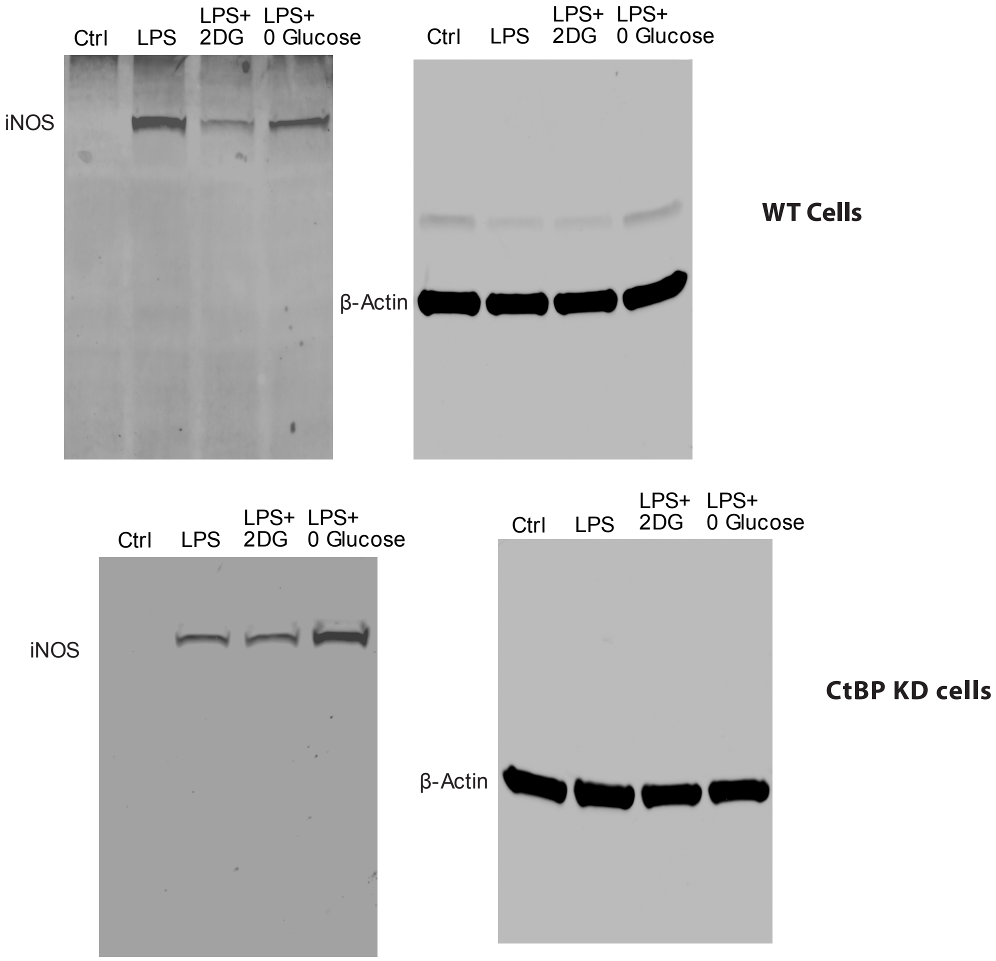

Full length immunoblots from Fig. 5B

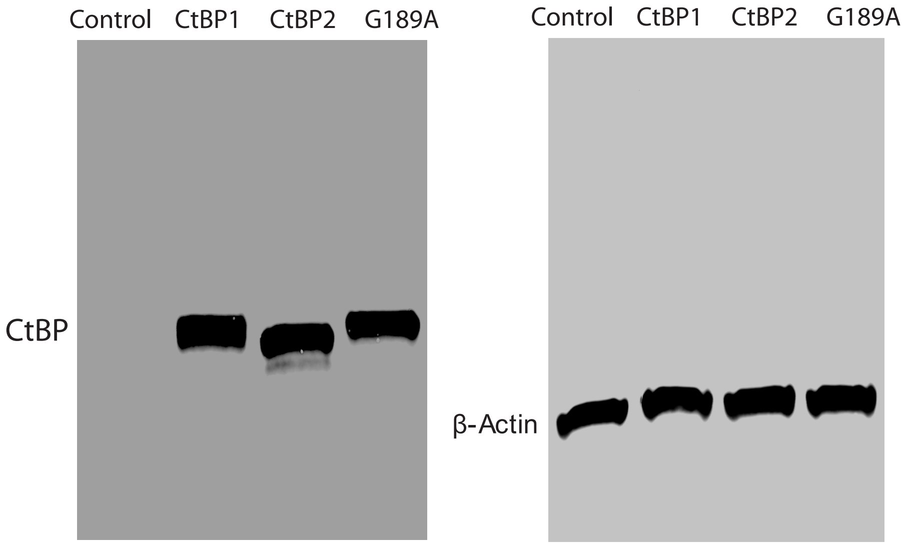

Full length immunoblots from Fig. 6A

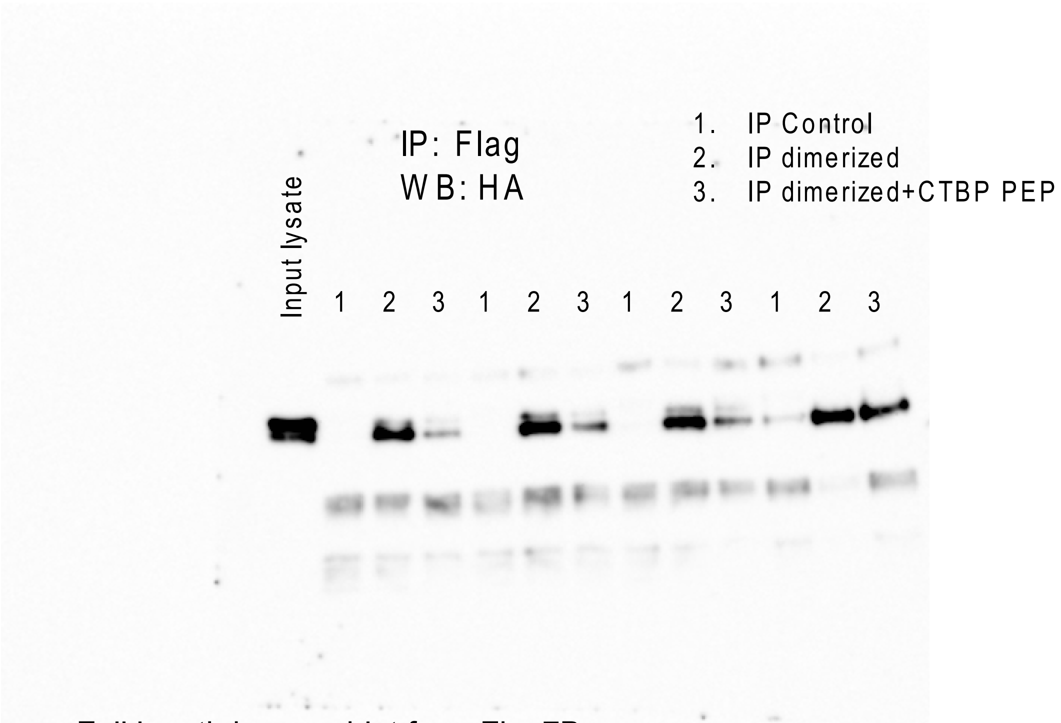

Full length immunoblot from Fig. 7B

Full length immunoblots from Fig. 8B

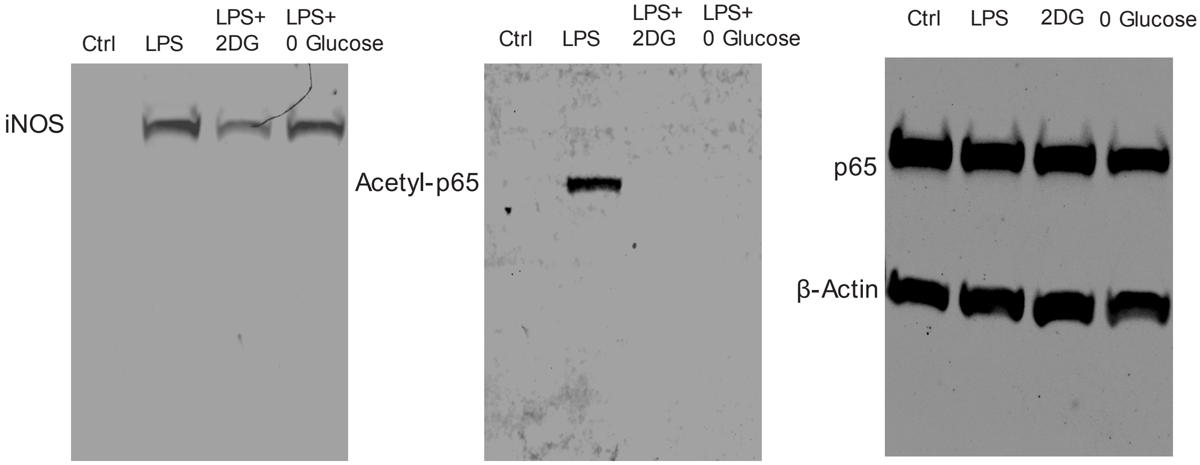

Full length immunoblots from Fig. 8D

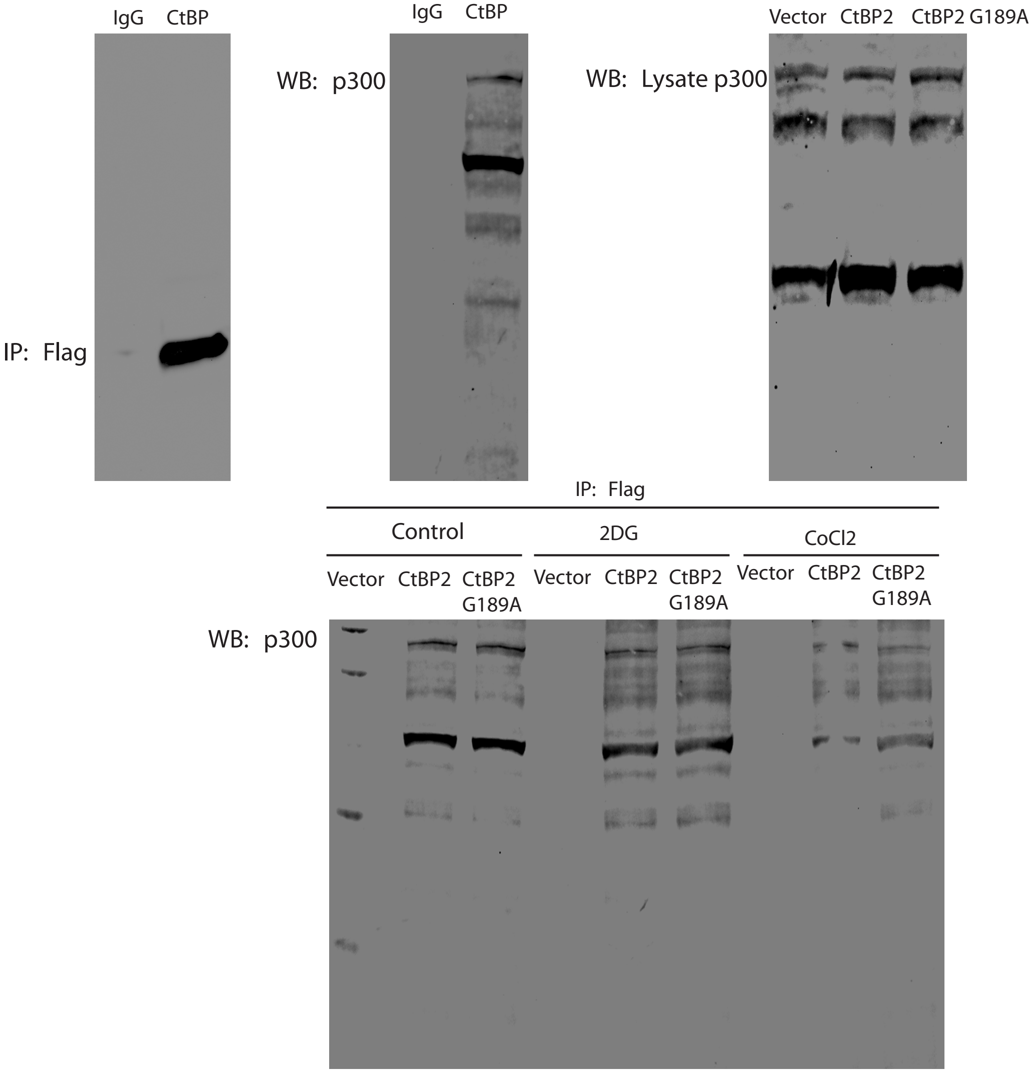

Supplement: Supplementary file 1 — Supplementary information [file 41467_2017_707_MOESM1_ESM.pdf]
